# Occluded Pedestrian-Attribute Recognition for Video Sensors Using Group Sparsity

**DOI:** 10.3390/s22176626

**Published:** 2022-09-01

**Authors:** Geonu Lee, Kimin Yun, Jungchan Cho

**Affiliations:** 1 College of Information Technology, Gachon University, Sengnam 13120, Korea; 2 Artificial Intelligence Research Laboratory, Electronics and Telecommunications Research Institute, Daejeon 34129, Korea

**Keywords:** deep learning, group-sparsity loss, temporal attention module, video-based pedestrian-attribute recognition

## Abstract

Pedestrians are often obstructed by other objects or people in real-world vision sensors. These obstacles make pedestrian-attribute recognition (PAR) difficult; hence, occlusion processing for visual sensing is a key issue in PAR. To address this problem, we first formulate the identification of non-occluded frames as temporal attention based on the sparsity of a crowded video. In other words, a model for PAR is guided to prevent paying attention to the occluded frame. However, we deduced that this approach cannot include a correlation between attributes when occlusion occurs. For example, “boots” and “shoe color” cannot be recognized simultaneously when the foot is invisible. To address the uncorrelated attention issue, we propose a novel temporal-attention module based on group sparsity. Group sparsity is applied across attention weights in correlated attributes. Accordingly, physically-adjacent pedestrian attributes are grouped, and the attention weights of a group are forced to focus on the same frames. Experimental results indicate that the proposed method achieved 1.18% and 6.21% higher $F_{1}$-scores than the advanced baseline method on the occlusion samples in DukeMTMC-VideoReID and MARS video-based PAR datasets, respectively.

## 1. Introduction

Pedestrian-attribute recognition (PAR) is a task that predicts various attributes of pedestrians detected by surveillance vision sensors, e.g., CCTV. It is a human-searchable semantic description and can be adopted in soft biometrics for visual surveillance [1]. Several studies have been conducted on this subject [2,3,4,5,6,7,8], owing to the importance of its applications, such as in finding missing persons and criminals. A few studies have focused on occlusion situations for pedestrian detection [9] and person re-identification [10,11,12] based on visual sensors. However, the occlusion problem in the field of PAR remains an open problem.

Due to the fact that other objects and persons obstruct pedestrians, it is impossible to resolve this challenge based on a single image. However, a video sensor contains more pedestrian information than an image, thus allowing a model to leverage information from multiple frames. Consider a case in which the lower body of a pedestrian is occluded in some frames but the other frames contain a visible lower-body appearance of the same pedestrian. In this case, we must use only the information obtained from the frame with the visible lower body rather than the one in which the lower body is occluded. Recently, Chen et al. [13] proposed a video-based PAR method that calculates temporal attention probabilities to focus on frames that are important for attribute recognition. However, this method concentrates on incorrect frames when a pedestrian is occluded by other objects or people.

**Figure 1:**
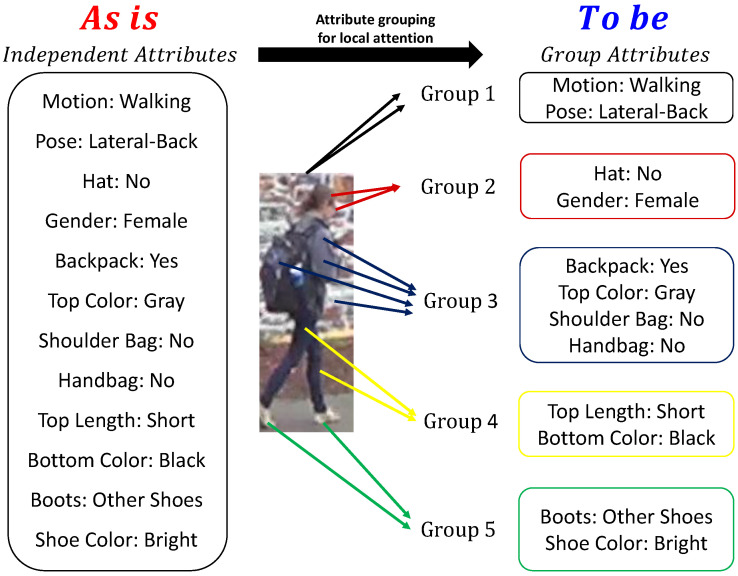
Attribute grouping for local attention. Physically-adjacent pedestrian attributes are grouped into one group. Group 1 is for attributes related to the entirety of a pedestrian. Groups 2, 3, 4, and 5 are for attributes related to the head, upper body, lower body, and feet of a pedestrian, respectively. The network focuses on the semantic information of the pedestrian such that it helps in recognizing pedestrian attributes occluded by obstacles.

Recent studies are yet to comprehensively consider occlusion analysis. In this study, we propose a novel method for improving PAR performance in occlusion cases. As an intuitive idea, to avoid concentrating on the frame with the occlusion, we select a frame that can best estimate each attribute. Therefore, one solution adopts the sparsity regularization [14] of temporal attention weights. In other words, sparse attention maximizes relevant information in the other weighted frames. However, our experimental results indicate that adding this simple sparsity constraint to the baseline method [13] does not accurately handle occlusion. This is because the method proposed in [13] employs multiple independent branches for multi-attribute classification. Sparsity-constrained temporal attention cannot understand the relationships between the attributes. However, pedestrian attributes are closely related to each other. In particular, semantically adjacent attributes exhibit more significant relationships, as illustrated in Figure 1. Therefore, the relationship between attributes is key to finding meaningless frames, and we formulate this relationship as temporal attention based on group sparsity.

**Figure 2:**
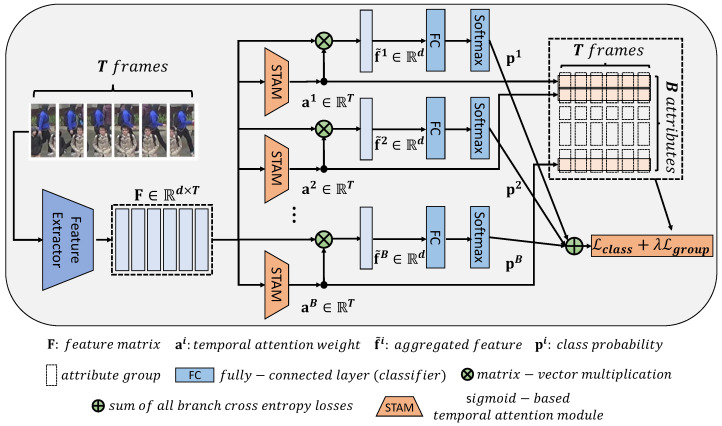
Overview of the network architecture of the proposed method. It comprises a feature extractor, Sigmoid-based temporal attention modules, and attribute classifiers. Due to the fact that the attributes of the pedestrians are closely related to each other, the attention weights for semantically adjacent attributes have similar values to each other, i.e., temporal frame attentions are not independent. To reflect this point, we formulate a temporal attention module based on the group-sparsity constraint. In the T×B block, the attention weights of the related attributes are grouped by the $L_{2}$ norm in each frame.

Group sparsity [15] is a more advanced method than sparsity; it can gather the related attention of the attributes into a single group. For instance, in Figure 1, information regarding “boots” and “shoe color" is destroyed at the same time an obstacle occludes the feet of a pedestrian. In this case, group sparsity categorizes the “boots” and “shoe color” together into one group. Then, their attention weights are simultaneously suppressed. Therefore, the group constraint achieves more improved results for occlusion situations than those of the sparsity method. Figure 2 presents an overview of the proposed method comprising a shared feature extractor, multiple attribute-classification branches, and a temporal attention module based on group sparsity across multiple branches.

Extensive experiments were conducted to demonstrate the improvement of the proposed method in its effectiveness against occlusion. The proposed method outperformed the advanced methods on the DukeMTMC-VideoReID [13,16,17] and MARS [13,18] benchmark datasets. In particular, the proposed method achieved 1.18% and 6.21% higher $F_{1}$-scores than those of the advanced baseline method on occlusion samples. We also validated the proposed method on additional occlusion scenarios with synthetic data, demonstrating that the proposed method consistently outperformed the advanced baseline method with a maximum $F_{1}$-score of 6.26%.

Our main contributions are summarized as follows.

- The proposed temporal attention module is designed to reflect the temporal sparsity of useful frames in a crowded video. Our model is guided to not pay attention to the occluded frame, but rather to the frame where relevant attributes are visible.
- When a pedestrian is occluded owing to obstacles, information on several related attributes is difficult to infer simultaneously. Therefore, we propose a novel group-sparsity-based temporal attention module. This module allows a model to robustly pay attention to meaningful frames to recognize the group attributes of a pedestrian.
- Extensive experiments provide performance analysis of PAR methods on various occlusion scenarios, where the proposed method outperformed the state-of-the-art methods.

The remainder of this paper is organized as follows. First, we introduce sparsity and group-sparsity regularizations, as well as other related work in Section 2.2, and then the proposed method is described in Section 3. Subsequently, Section 4 presents details on the implementation and experimental results. Finally, we discuss and conclude the paper in Section 5.

## 2. Preliminaries

### 2.1. Sparsity and Group-Sparsity Regularizations

In deep learning, training a classifier model *f* is an under-determined problem due to finite datasets [19]. A regularization term *R* is used to impose prior knowledge on parameters w as

(1) $$\min_{w}\sum_{i=1}^{n}L\left(f\left(x_{i};w\right),y_{i}\right)+\lambda R\left(w\right),$$

where $x_{i}$, *L*, and λ represents the *i*-th training example, a loss function between predicting results $f(x_{i};w)$ and their ground truths $y_{i}$, and a hyper-parameter that controls the importance of the regularization term, respectively.

**Sparsity Regularization** is adopted to induce the model to be sparse. The feasible constraint for sparsity is to reduce the number of nonzero parameter elements, defined as $L_{0}$ norm $R\left(w\right)={∥w∥}_{0}$. However, because the $L_{0}$ norm solution is NP-hard problem, the $L_{1}$ norm $R\left(w\right)={∥w∥}_{1}=\sum_{j}\left|w_{j}\right|$ is used to approximate $L_{0}$ norm in several deep learning problems [20].

**Group-sparsity regularization** is employed to introduce the *K*-group structure into the leaning problem as $R\left(w\right)={\left||w|\right|}_{2,1}=\sum_{k=1}^{K}{∥w^{k}∥}_{2}$, where $∥w^{k}{∥}_{2}=\sqrt{\sum_{j=1}^{|G^{k}|}{\left(w_{j}^{k}\right)}^{2}}$. This is interpreted as imposing an $L_{2}$ norm regularizer on members of each group, $w^{k}\in R^{|G^{k}|}$, and then inducing an $L_{1}$ norm over groups [21,22].

**Applications:** Nguyen et al. [20] proposed a sparse temporal pooling network for action localization in a video. Unlike the sparsity loss method that adjusts each value, the group-sparsity loss method simultaneously controls the values associated with each other [21,22,23,24]. We propose a method that simultaneously adjusts the attention weights of pedestrian attributes by designing the group-sparsity constraint.

### 2.2. Pedestrian-Attribute Recognition

**Video-based PAR:** Chen et al. [13] proposed an attention module that indicates the extent to which the model pays attention to each frame for each attribute. They designed branches and classifiers for each attribute in the video. Specker et al. [25] employed global features before temporal pooling to utilize the different pieces of information from various frames. However, existing video-based PAR methods are yet to comprehensively consider the occlusion problem. In this study, we focus on the occlusion handling of video-based PAR.

**Image-based PAR:** Liu et al. [2] proposed the HydraPlus-Net network that utilizes multi-scale features. Tang et al. [26] proposed an attribute localization module (ALM) that learns specific regions for each attribute generated from multiple levels. Furthermore, Ji et al. [27] proposed a multiple-time-steps attention mechanism that considers the current, previous, and next time steps to understand the complex relationships between attributes and images. Jia et al. [28] proposed Spatial and Semantic Consistency Regularizations (SSC${}_{soft}$). The spatial consistency regularization understands the regions related to each attribute. In addition, they proposed a semantic consistency regularization to extract the unique semantic features of each attribute.

With image-based PAR, it is difficult to achieve accurate attribute recognition for various situations, such as occlusion situations. On the other hand, videos contain more information than images; recently, the number of video-based studies has been increasing.

## 3. Proposed Method

### 3.1. Problem Formulation

**Figure 3:**
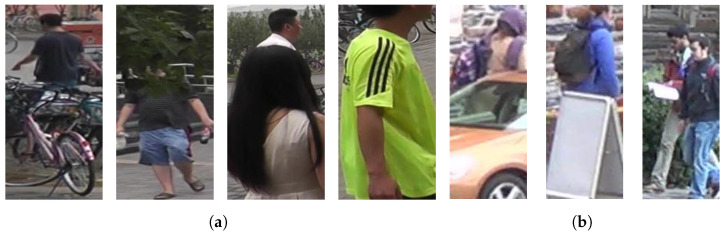
(**a**,**b**) represent the occlusion types in MARS and DukeMTMC-VideoReID datasets, respectively. Various occlusion types exist, such as a lower body or head of a pedestrian occluded by other pedestrians, tracking failure, and so forth.

Figure 3 presents examples of occluded pedestrian images from two video PAR datasets (DukeMTMC-VideoReID and MARS [13]). Typically, pedestrian images obtained from surveillance cameras in the real world are often obscured by crowds of people, cars, and buildings. In addition, the instability of pedestrian tracking results in distorted pedestrian images. Therefore, it is important to correctly recognize pedestrian attributes in occlusion situations; however, occluded pedestrian images make it impossible to obtain single-image-based PAR. This study attempts to achieve improved PAR using multiple frames, i.e., video-based PAR.

### 3.2. Overview

The proposed method comprises a feature extractor, attention modules, and attribute classifiers. In addition, the inputs are a set of *T* frames, as illustrated in Figure 2.

First, any feature-extraction network can be used. Here, we utilize the same feature extractor employed in our baseline [13], which comprises a ResNet [29] and two convolution modules, to extract two types of features according to their relevance to the identification (for more details, please refer to [13]). It returns a feature matrix $F\in R^{d\times T}$ that contains a set of *d*-dimensional feature vectors corresponding to *T* frames as $F=[f_{1},f_{2},\ldots ,f_{T}]$. However, the body parts of a pedestrian are often occluded, owing to obstacles and other pedestrians in actual videos. Therefore, the information required to recognize pedestrian attributes differs for each frame, even in the same video.

Second, the proposed network includes a temporal attention module for aggregating multiple frames that is implemented by multiplying the feature matrix F as

(2) $${\tilde{f}}^{i}=Fa^{i}=\sum_{t=1}^{T}a_{t}^{i}\cdot f_{t}^{i},$$

where ${\tilde{f}}^{i}\in R^{d}$ is an aggregated feature vector, while $a^{i}$ is an attention-weight vector obtained by the temporal attention module in Section 3.3. The superscript *i* indicates the *i*-th attribute type (e.g., hat, backpack, shoe type, and color).

Finally, multi-branch classifiers are employed for multi-labeled attribute classifications as depicted in Figure 2. Notably, unlike the existing work [13], which trains multiple attribute classifiers by solely adopting independent classification losses, the proposed method reliably trains multiple classifiers using feature vectors constrained by a temporal attention module based on group sparsity.

In the following sections, we will explain the novel temporal attention module based on group sparsity.

### 3.3. Temporal-Attention-Module-Based Classification

**Figure 4:**
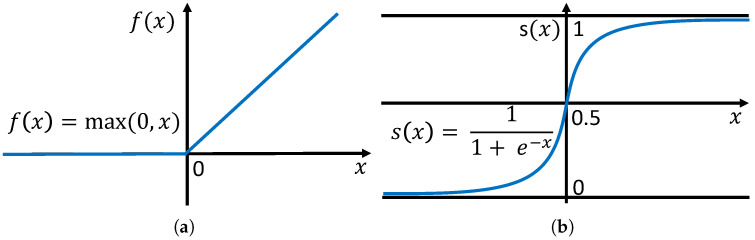
Activation functions. (**a**) ReLU function; (**b**) Sigmoid function.

Chen et al. [13] designed the temporal attention as a Softmax-based probabilistic temporal attention module (PTAM) that calculates important probabilities for frames in the temporal direction and returns an attention weight vector $a\in R^{T}$. However, PTAM comprises Conv-ReLU-Conv-ReLU-Softmax. ReLU [30] converts all the negative values to 0 as illustrated in Figure 4a, while Softmax normalizes the sum of the attention weights of the *T* frame equal to 1, i.e., $Softmax\left(a\right)=\left[\frac{e^{a_{1}}}{\sum_{j=1}^{T}e^{a_{j}}},\frac{e^{a_{2}}}{\sum_{j=1}^{T}e^{a_{j}}},\ldots ,\frac{e^{a_{T}}}{\sum_{j=1}^{T}e^{a_{j}}}\right]$. This makes it difficult to obtain attention weights that reflect the sparsity constraints [20]. In other words, if the weight of a particular frame becomes 1, the weight of the rest of the frame becomes 0. This is not optimal, as the weights of several frames should have high values. To address this issue, Ref. [20] adopted the Sigmoid-based attention module. Inspired by [20], we use a Sigmoid-based temporal attention module (STAM) configured with Conv-ReLU-Conv-Sigmoid. The Sigmoid after Conv allows any frame to have a weight close to 0 or 1, as illustrated in Figure 4b.

In multi-branch cases, a temporal-attention-weight vector for the *i*-th attribute type, $a^{i}\in R^{T}$, can be obtained as

(3) $$a^{i}=STAM^{i}\left(F\right).$$

Finally, an aggregated feature vector for the *i*-th attitude classification, ${\tilde{f}}^{i}\in R^{d}$, is obtained by Equation (2). Subsequently, we pass ${\tilde{f}}^{i}$ to the *i*-th linear attribute classifier, and a prediction vector $p^{i}$ is obtained for each attribute as:

(4) $$p^{i}=Softmax\left(W^{i}{\tilde{f}}^{i}\right),$$

where $W^{i}\in R^{c_{i}\times d}$ represents a weight matrix of a fully connected layer for the *i*-th attribute classification branch, and $c_{i}$ denotes the number of classes of the branch. The classification loss $L_{class}$ is the sum of the cross-entropy (CE) [31] of the attributes.

(5) $$L_{class}=\sum_{i=1}^{B}\beta^{i}CE\left(p^{i}\right),$$

where *B* denotes the number of branches for each attribute in Figure 2. $\beta^{i}$ is a balancing hyperparameter for the *i*-th attribute classification. It is set as a reciprocal of the number of classes in each attribute, because each attribute classification has a different number of classes.

### 3.4. Limitation of Sparsity Constraint on STAM

The temporal attention weight $a^{i}$ in Equation (2) is an indicator that represents the importance of each frame. The sparsity constraint for the attention weight is used to improve the importance indication of frames and is computed by the $L_{1}$ norm on $a^{i}$.

(6) $$L_{sparsity}=\sum_{i=1}^{B}{∥a^{i}∥}_{1},$$

where *B* denotes the number of branches of each attribute. The sparsity loss is the operation of the $L_{1}$ norm per branch of each attribute. From the formulation, the sparsity constraint is expected to have the effect of selecting frames that are not occluded from *T* frames independently for each branch.

However, compared with the baselines, our experimental results, presented in Section 4, indicate that the sparsity constraint on the STAM fails to assign importance to the correct frame, thereby degrading the PAR performance sometimes.

*Why does the sparsity constraint fail to improve the overall performance?*

**Figure 5:**
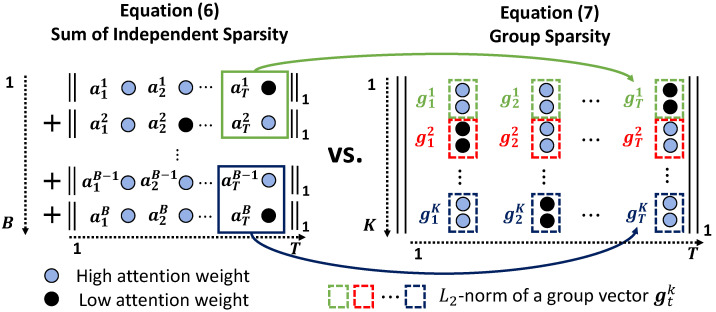
Comparison between the sparsity- and group-sparsity-based constraints. Unlike the sparsity-based method that adjusts each value independently, the group-sparsity-based method simultaneously controls the values associated with each other.

As illustrated on the left-hand side of Figure 5, the sparsity constraint on STAM is independently applied to the temporal attention weights by the $L_{1}$ norm for each branch; hence, the attention weights of each branch solely depend on the temporal information in each attribute. This implies that the sparsity constraint does not help a model understand the relationship between each attribute. However, pedestrian attributes are closely related to each other. As presented in Figure 3, information about some attributes, such as the type and color of the bottom and shoe of a pedestrian, respectively, is damaged simultaneously if a the lower body or feet of the pedestrian is/are occluded. Therefore, another constraint is required to guide the model to understand the relationship between pedestrian attributes, which is important for achieving improved performance, by considering various occlusion situations. In the next section, we design the attribute relationships as attribute groups and formulate the group constraints of these attributes.

### 3.5. Group-Sparsity Constraint on STAM

Group sparsity extends and generalizes how to learn the correct sparsity regularization by which prior assumptions on the structure of the input variables can be incorporated [15]. Regarding the attributes of an occluded pedestrian, the prior assumption is that these attributes can be partitioned into *K* groups based on their relevance, i.e., $G^{k}$ where k=1,2,…,K, as illustrated in Figure 1. Accordingly, the attention weights in the same group at time *t*, $\left\{a_{t}^{i}|i\in G^{k}\right\}$, can be constrained by considering the group structure.

The method for grouping multiple attribute weights at time *t* involves introducing a new vector at time *t* using each attribute group, i.e., $g_{t}^{k}\in R^{|G^{k}|}$, as presented on the right-hand side of Figure 5. By summing the $L_{2}$ norm of a group vector $g_{t}^{k}$, we can define two sparsity constraints on attributes and time as

(7) $$L_{group}=\sum_{t=1}^{T}\sum_{k=1}^{K}\gamma_{k}{∥g_{t}^{k}∥}_{2},$$

where ${∥g_{t}^{k}∥}_{2}$ always has positive values; hence, the sum of these values has the same effect as the $L_{1}$ norm [21,22,23]. $\gamma_{k}$ is a balancing hyperparameter for the *k*-th group in the sum of all the group-sparsity loss functions. It is set as a reciprocal of the number of attributes in each group, because each group has a different number of attributes.

The $L_{group}$ constraint on STAM simultaneously increases or decreases the attention weights of specific groups in particular frames. This helps a model understand the frames that are more important for each group, including the groups that are recognizable in the same frame. This constraint is consistent with the prior assumption that groups exist between attributes. In addition, it does not employ explicit local patches in frames for the recognition of specific attributes. It adopts implicit attention via attribute groups, thereby enabling improved attribute recognition for pedestrian appearance distortions due to tracking failures.

Finally, the total loss function comprises $L_{class}$ and $L_{group}$, described above, as follows:

(8) $$L_{total}=L_{class}+\lambda L_{group},$$

where λ represents a weight factor that combines the classification and group-sparsity losses.

## 4. Experiments

### 4.1. Implementation Details

Table 1 presents the attribute groups of the group sparsity for the experiments. We employed the same feature extractor as [13], pretrained on the ImageNet dataset [32]. The initial learning rate was set to $3\times 10^{-4}$ and multiplied by 0.3 at 100 epochs. The weight decay was set to $5\times 10^{-4}$ for the Adam optimizer [33]. For the input, the width and height of the frame were resized to 112 and 224 pixels, respectively. The weight factor λ in Equation (8) was set to 0.02. The batch size for training was set to 64. The model was trained for 200 epochs, and the best results among the measurements were reported every 20 epochs. The sequence length *T* of the consecutive and non-overlapping frames for training was set to 6, according to a previous study [13]. In the test phase, we divided the trajectory of a pedestrian into segments comprising 6 frames. The divided segments were independently inferred, and the results were averaged for PAR. In other words, performance was measured using one prediction per trajectory, according to [13]. We utilized a single NVIDIA Titan RTX GPU for both training and inference. Regarding our experimental setting, in the absence of an additional explanation, we follow the process detailed in the baselines [13] for a fair comparison. The random seed for the experiments was fixed deterministically.

### 4.2. Evaluation Metrics and Datasets

We evaluated the proposed method using the average accuracy and $F_{1}$-score that decrease when the algorithm fails to recognize the correct pedestrian attributes. For the extensive experiments, we used two video-based PAR datasets: DukeMTMC-VideoReID and MARS [13], which were derived from the reidentification datasets, DukeMTMC-VideoReID [16] and MARS [18], respectively. Chen et al. [13] reannotated them for the video-based PAR datasets.

#### 4.2.1. DukeMTMC-VideoReID Dataset

The DukeMTMC-VideoReID dataset contains 12 types of pedestrian-attribute annotations. Eight of these attributes are binary types: backpack, shoulder bag, handbag, boots, gender, hat, shoe color, and top length. The other four attributes are multi-class types: motion (walking, running, riding, staying, various), pose (frontal, lateral-frontal, lateral, lateral-back, back, various), bottom color (black, white, red, gray, blue, green, brown, complex), and top color (black, white, red, purple, gray, blue, green, brown, complex). The attributes were annotated per trajectory and the total number of trajectories was 4832. We excluded four trajectories with fewer frames than the segment length *T*, while the remaining 4828 trajectories were adopted in the experiments. For the training, 2195 trajectories were used, 413 of which contained occlusions, as illustrated in Figure 3b. For the test, 2633 trajectories were employed, 449 of which contained occlusions. The average length of the trajectories was approximately 169 frames.

#### 4.2.2. MARS Dataset

The MARS dataset contains 14 types of pedestrian-attribute annotations. Ten of these attributes are binary types: shoulder bag, gender, hair, bottom type, bottom length, top length, backpack, age, hat, and handbag. The other four attributes are multi-class types: motion (walking, running, riding, staying, various), pose (frontal, lateral-frontal, lateral, lateral-back, back, various), top color (black, purple, green, blue, gray, white, yellow, red, complex), and bottom color (white, purple, black, green, gray, pink, yellow, blue, brown, complex). The attributes were also annotated per trajectory, and the total number of trajectories was 16,360. We also excluded five trajectories with fewer frames than the segment length *T*, and the remaining trajectories were 16,355. For the training, 8297 trajectories were used, 35 of which contained occlusions, as illustrated in Figure 3a. For the test, 8058 trajectories were used, 30 of which contained occlusions. The average length of the trajectories was approximately 60 frames.

### 4.3. Comparisons with State-of-the-Art Methods

The proposed method was compared with five baselines: Chen et al. [13], 3D-CNN [34], CNN-RNN [35], ALM [26], and SSC${}_{soft}$ [28]. The Chen et al. [13] method is a state-of-the-art video-based PAR method. CNN-RNN and 3D-CNN are video-based PAR methods compared in [13]. ALM [26] and SSC${}_{soft}$ [28] are two state-of-the-arts for image-based PAR. For fair comparisons, we adopted the average values for each image of trajectories to evaluate the ALM and SSC${}_{soft}$ methods on video-based datasets. We retrained the ALM [26] using the officially published code. For SSC${}_{soft}$ [28], we re-implemented it because there is no official code. In the case of ALM [26] and SSC${}_{soft}$ [28], the image batch size was set to 96, and the learning rate was adjusted to 7.5 × 10${}^{-5}$, according to [36].

To evaluate the improvement of the proposed method in occlusion situations, we compared its performance with those of the baselines by only adopting the occlusion samples. Table 2 presents the results on the DukeMTMC-VideoReID and MARS datasets. To ensure accurate evaluation, we excluded the “hat” and “handbag” attributes of the MARS dataset when evaluating all methods, because the ground truth of both attributes for all occlusion samples was the same, i.e., “no”. As presented in Table 2, the proposed method outperformed the baselines in all cases and achieved average accuracies of 88.36% and 71.94%, including average $F_{1}$-scores of 70.21% and 61.88% on the occlusion samples of the DukeMTMC-VideoReID and MARS datasets, respectively. In particular, the proposed method achieves superior performance over the state-of-the-art ALM [26] and SSC${}_{soft}$ [28] methods, which extended to video using multi-frame averages. This shows that the image-based PAR methods have limitations in effectively using multiple frames when extended to video. In the real world, pedestrians are often occluded by various environments, so performance improvement of the proposed method in occlusive situations is not trivial.

To verify that the proposed method does not have severe negative effects on non-occlusion samples, we also evaluated its performance using total samples, including the occlusion and non-occlusion samples. Table 3 presents the performances of the methods on the total samples of the DukeMTMC-VideoReID and MARS datasets, where the proposed method outperformed the baselines. The Chen et al. [13] method exhibited a slightly better average accuracy in just one case, in the DukeMTMC-VideoReID dataset. However, because the measure of average accuracy did not consider a data imbalance, the difference was negligible. For instance, if there are 90 negative samples and 10 positive samples among the 100 total samples, the model can obtain high accuracy by predicting most of the samples as being negative, e.g., when true negative, true positive, false negative, and false positive are 90, 1, 9, and 0, respectively, the accuracy is 91%, and the $F_{1}$-score is 18.18%. Therefore, the average $F_{1}$-score is a better measure than the average accuracy for imbalanced datasets.

### 4.4. Ablation Study

#### 4.4.1. Effects of the Weight Factor λ

We compared the experimental results according to the weight factor λ in Equation (8). The weight factor λ is a parameter that adjusts sparsity. As presented in Table 4, the proposed method exhibits higher $F_{1}$-scores than those of the baseline methods, regardless of the λ values, and the best results were obtained with λ=0.02.

#### 4.4.2. Comparisons between PTAM and STAM

We analyzed PTAM and STAM by applying them along with each method. Table 5 demonstrates that sparsity has the worst performance for occlusion samples in terms of both accuracy and $F_{1}$-scores. As explained in Section 3.4, the sparsity constraint cannot help a model understand the relationship between attributes. However, the proposed method using the group-sparsity-constrained STAM, which understands the relationship between each attribute, exhibited the best performance among the other methods.

### 4.5. Qualitative Results

**Figure 6:**
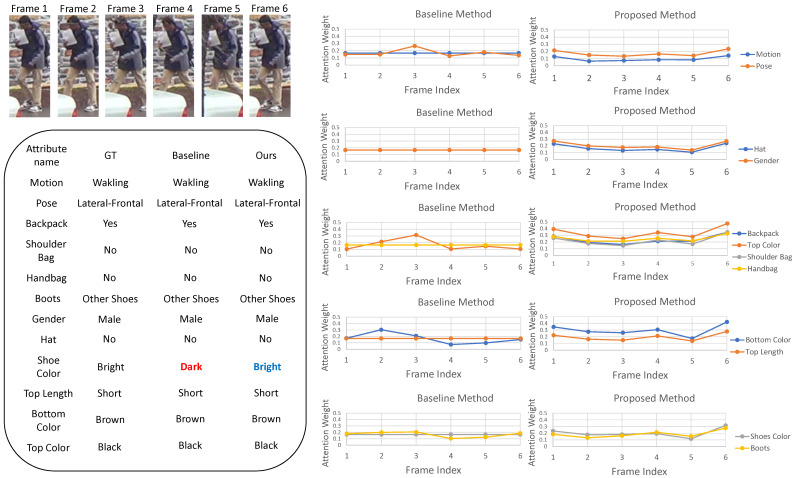
Qualitative results for the DukeMTMC-VideoReID dataset. It presents the attention weights of the group attributes and PAR results. For the groups related to the lower body, the proposed method has low attention weights in the occluded frames. However, the attention weights of the baseline method (Chen et al. [13]) are almost the same in all the frames.

We visualized the temporal attention weight vector with various segment frames to analyze the improvement of the proposed method in occlusion situations. Figure 6 presents the temporal attention vectors and PAR results of the method presented by Chen et al. [13] and that of our method for all the groups in the DukeMTMC-VideoReID dataset. The values of the baseline method are similar in all the frames. Thereby, the baseline method failed to recognize the “shoe color” attribute. In contrast, the values of the proposed method are different in each frame. Moreover, the values of the occlusion frames are lower than those of the general frames. The attention weights of the bottom- and top-length attributes are simultaneously controlled, because they belong to the same group. For the same reason, the attention weights of the “shoe color” and “boot” attributes are also simultaneously adjusted. Consequently, the proposed method accurately predicted all attributes. It shows that the proposed group-sparsity constraint helps STAM accurately focus on non-occlusion frames.

### 4.6. Evaluation of Additional Occlusion Scenarios

**Figure 7:**
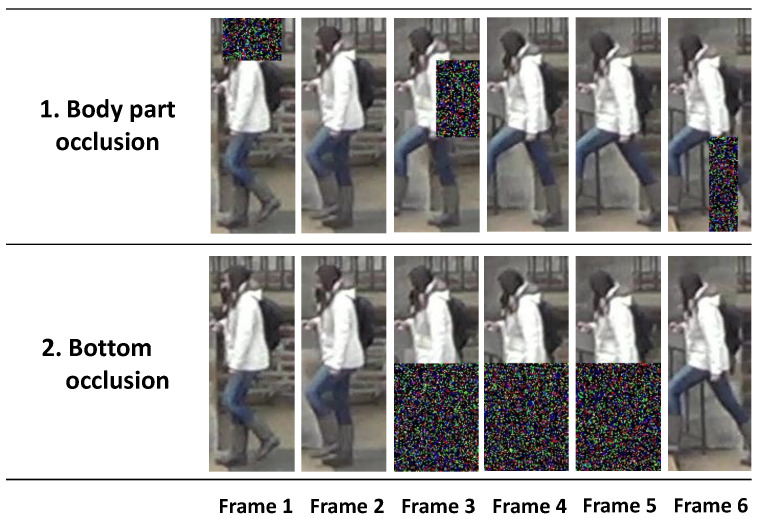
Examples of two occlusion scenarios.

We designed two synthetic occlusion scenarios, as illustrated in Figure 7, to validate the improvement of the proposed method on several occlusion samples. These two occlusion scenarios are designed to analyze the impact on recognition performance if a part of the appearance of pedestrian frames is distorted by blurring, low illumination, or an object such as another pedestrian or car.

The first scenario was a body-part occlusion. In this scenario, we randomly selected three frames among the segment frames. Subsequently, the head of a pedestrian and the left and right sides of their upper and lower body, respectively, were randomly occluded. The second scenario was the bottom occlusion scenario that simulated a situation in which cars and bicycles passed through and occluded the lower body of the pedestrian. We randomly selected three consecutive frames.

In the process of constructing the scenarios, we did not apply the additional occlusion situations to real occlusion samples in the datasets. The number of test samples for each scenario was 2633 and 8058 for the DukeMTMC-VideoReID and MARS datasets, respectively, which are the same as the total number of test samples in the original datasets.

We did not retrain the baseline and proposed methods to prevent the models from learning the tendency of synthetic occlusion. We used the same models in Section 4.3 and Section 4.4 and evaluated them on two scenario samples. Table 6 presents the results for the body-part and bottom occlusion scenarios. In all cases, the proposed method achieved better results than those of the baseline methods. Table 7 shows the average $F_{1}$-scores according to the number of consecutive occlusion frames on the bottom occlusion scenario samples of the DukeMTMC-VideoReID and MARS datasets. As the number of consecutive occlusion frames increases, the amount of information for recognizing attributes decreases, and, thus, the performances of all methods were degraded. Nevertheless, the proposed method consistently achieved better average $F_{1}$-scores in comparison to those of the baselines as the number of consecutive occlusion frames increased. The obtained experimental results indicate that the proposed method is effective in handling occlusions, regardless of the scenario. Accordingly, we can conclude that the proposed method is more suitable for real-world scenarios with many occlusions than the compared methods.

## 5. Conclusions and Future Work

This study proposed a novel video-based PAR method to improve PAR in various occlusion situations. The proposed method was formulated as a group sparsity to consider the relationship between pedestrian attributes. In addition to improving the temporal attention weights for non-occluded frames, it exhibited the effect of simultaneously excluding multiple occluded attributes by understanding the relationship between each attribute within the frame. In other words, the proposed method focused more on information about attributes that were not occluded and related to each other in the specific frames.

The proposed method was designed to improve PAR in occlusion situations; however, only a few datasets contained sufficient occlusion samples. To address this limitation, the proposed method was also validated on additional scenarios with synthetic samples. The results obtained from extensive experiments demonstrate that the proposed method consistently outperformed most of the baselines. In the future, we will study how to generate an extensive and natural occlusion situation. Furthermore, we will investigate a one-stage method that can detect and track pedestrians and better recognize pedestrian attributes in an extensive occlusion situation.

## Figures and Tables

**Table 1:** Attribute groups for DukeMTMC-VideoReID and MARS datasets.

Group	DukeMTMC-VideoREID	MARS
Whole	motion, pose	motion, pose
Head	hat, gender	age, hat, hair, gender
Upper Body	backpack, top color, shoulder bag,	backpack, top color, shoulder bag,
	handbag	handbag, top length
Lower Body	top length, bottom color	bottom length, bottom color,
		type of bottom
Foot	boots, shoe color	-

**Table 2:** Comparisons of the results obtained for the occlusion samples of the DukeMTMC-VideoReID and MARS datasets. The **bold** indicates the best result.

Dataset	Method	Average	Average
		Accuracy (%)	$F_{1}$-Score (%)
DukeMTMC -VideoReID	Chen et al. [13]	88.33	69.03
	3DCNN [34]	84.41	61.38
	CNN-RNN [35]	87.94	68.12
	ALM [26]	86.99	65.87
	SSC${}_{soft}$ [28]	86.86	65.01
	Ours	**88.36**	**70.21**
MARS	Chen et al. [13]	66.39	55.67
	3DCNN [34]	60.83	46.16
	CNN-RNN [35]	65.83	53.79
	ALM [26]	67.50	55.73
	SSC${}_{soft}$ [28]	68.89	57.44
	Ours	**71.94**	**61.88**

**Table 3:** Comparisons of the results for the total samples of the DukeMTMC-VideoReID and MARS datasets. The **bold** indicates the best result.

Dataset	Method	Average	Average
		Accuracy (%)	$F_{1}$-Score (%)
DukeMTMC -VideoReID	Chen et al. [13]	**89.12**	71.58
	3DCNN [34]	85.38	64.66
	CNN-RNN [35]	88.80	71.73
	ALM [26]	88.13	69.66
	SSC${}_{soft}$ [28]	87.52	68.71
	Ours	88.98	**72.30**
MARS	Chen et al. [13]	86.42	69.92
	3DCNN [34]	81.96	60.39
	CNN-RNN [35]	86.49	69.89
	ALM [26]	86.56	68.89
	SSC${}_{soft}$ [28]	86.01	68.15
	Ours	**86.75**	**70.42**

**Table 4:** Analysis of the group-sparsity loss for the occlusion samples of the DukeMTMC-VideoReID and MARS datasets. The **bold** indicates the best result.

Dataset	Method	Average	Average
		Accuracy (%)	$F_{1}$-Score (%)
DukeMTMC -VideoReID	Chen et al. [13]	88.33	69.03
	λ = 0.005	**88.38**	69.85
	λ = 0.03	88.16	69.62
	λ = 0.02	88.36	**70.21**
MARS	Chen et al. [13]	66.39	55.67
	λ = 0.005	68.06	55.07
	λ = 0.03	70.00	58.89
	λ = 0.02	**71.94**	**61.88**

**Table 5:** Comparisons between the sparsity-based and group-sparsity-based (ours) constraints for the occlusion samples of the DukeMTMC-VideoReID and MARS datasets. The **bold** indicates the best result.

Dataset	Method	PTAM	STAM	Average	Average
				Accuracy (%)	$F_{1}$-Score (%)
DukeMTMC -VideoReID	Chen et al. [13]	✓	-	88.33	69.03
	Sparsity	✓	-	87.99	69.05
	Group sparsity	✓	-	88.23	**70.24**
	Chen et al. [13]	-	✓	87.94	69.26
	Sparsity	-	✓	87.68	67.52
	Group sparsity	-	✓	**88.36**	70.21
MARS	Chen et al. [13]	✓	-	66.39	55.67
	Sparsity	✓	-	70.00	57.76
	Group sparsity	✓	-	**71.94**	61.70
	Chen et al. [13]	-	✓	66.94	55.92
	Sparsity	-	✓	69.17	57.80
	Group sparsity	-	✓	**71.94**	**61.88**

**Table 6:** Comparisons of the results for the two occlusion scenarios of the DukeMTMC-VideoReID and MARS datasets. The **bold** indicates the best result.

Dataset	Method	Body Part		Bottom	
		Average Accuracy (%)	Average $F_{1}$-Score (%)	Average Accuracy (%)	Average $F_{1}$-Score (%)
DukeMTMC -VideoReID	Chen et al. [13]	88.67	70.94	87.03	66.85
	3DCNN [34]	85.31	63.99	82.28	58.40
	CNN-RNN [35]	88.73	71.17	88.50	70.00
	ALM [26]	88.08	69.45	87.17	66.98
	SSC${}_{soft}$ [28]	87.60	67.87	86.60	65.64
	Ours	**88.95**	**71.97**	**88.59**	**70.66**
MARS	Chen et al. [13]	85.97	68.34	82.79	62.55
	3DCNN [34]	81.64	59.42	79.05	55.58
	CNN-RNN [35]	86.42	69.49	85.95	68.34
	ALM [26]	86.32	67.87	85.77	65.96
	SSC${}_{soft}$ [28]	85.34	65.18	84.61	63.95
	Ours	**86.73**	**70.05**	**86.08**	**68.81**

**Table 7:** Comparisons of the average $F_{1}$-scores (%) for according to the number of consecutive occluded frames on the bottom occlusion scenario of the DukeMTMC-VideoReID and MARS datasets. The **bold** indicates the best result.

Dataset	# Consecutive Occlusion Frames	Chen et al. [13]	3DCNN [34]	CNN-RNN [35]	ALM [26]	SSC${}_{\mathrm{soft}}$ [28]	Ours
DukeMTMC -VideoReID	1	70.79	63.46	71.15	69.18	68.00	**72.04**
	2	69.63	61.15	70.38	68.37	66.98	**71.61**
	3	66.85	58.40	70.00	66.98	65.64	**70.66**
	4	61.28	55.77	67.44	64.78	63.68	**68.16**
	5	55.94	54.22	**63.77**	62.03	61.16	63.54
MARS	1	68.37	59.13	69.59	68.40	67.28	**70.19**
	2	66.11	57.38	69.09	67.62	65.50	**69.69**
	3	62.55	55.58	68.34	65.96	63.95	**68.81**
	4	57.48	54.14	67.01	64.30	61.84	**67.62**
	5	51.50	52.97	65.04	62.18	59.13	**65.67**

## Data Availability

The dataset information. DukeMTMC-VideoReID: https://github.com/Yu-Wu/DukeMTMC-VideoReID MARS: http://zheng-lab.cecs.anu.edu.au/Project/project_mars.html (accessed on 20 July 2022).
